# Detection of Fungi in Indoor Environments and Fungus-Specific IgE Sensitization in Allergic Children

**DOI:** 10.1097/WOX.0b013e3181ba7daf

**Published:** 2009-09-15

**Authors:** Mitsuhiko Nambu, Hisashi Kouno, Maki Aihara-Tanaka, Hideharu Shirai, Kosuke Takatori

**Affiliations:** 1 Department of Pediatrics, Tenri, Nara, Japan; 2 Department of Clinical Pathology, Tenri Hospital, Tenri, Nara, Japan; 3 Division of Microbiology, National Institute of Health Sciences, Tokyo, Japan; 4 Institute of Tokyo Environmental Allergy, Tokyo, Japan

**Keywords:** fungus, *Alternaria*, IgE, house care

## Abstract

**Background:**

The aim of this study is to investigate relationships between fungal colonization in the house and IgE sensitization to fungi, and to clarify the effects of house care in relation to fungi.

**Materials and Methods:**

We measured levels of fungi in the houses of 52 allergic children. Of these, 32 children displayed detectable levels of IgE (≥ 0.35 U_A_/ml) to a combination of fungi (positive group). The remaining 20 children were not sensitized to fungi (negative group). Each fungus-specific IgE level was also measured in sera of the positive group, and a questionnaire-based survey was conducted for daily lifestyles.

**Results:**

*Cladosporium *was the most prevalent in the houses. From the 32 sera of the positive group, specific IgE levels ≥ 0.70 U_A_/ml were most frequently detected in 21 sera for *Alternaria*. Children in whose houses *Alternaria *was found displayed higher levels of *Alternaria*-IgE than those in whose houses where *Alternaria *was not found. In addition, *Alternaria*-IgE level was lower for children using an air purifier than for children who were not. Windows were more frequently opened for ventilation in negative-group houses than in positive-group houses.

**Conclusions:**

The existence of *Alternaria *might strongly induce IgE sensitization for *Alternaria*. Using an air purifier and frequently opening windows may minimize fungal sensitization of allergic children.

## 

In addition to house dust mites (HDMs), pet dandruff, and pollens, fungi represent important allergens [1-7]. Several studies of environmental interventions for allergic patients have recently been reported, [8-11] but the effectiveness of interventions was only on HDM allergen levels, and fungal indoor contamination was not investigated in those studies. In our previous study, a HDM-free pillow could not perfectly block fungi to penetrate inside [12]. As for fungi Immunoglobulin E (IgE) sensitization, atopic patients often have detectable levels of serum IgE to fungi, and cross-reactivity among different fungal species has been recently reported to be important for fungal sensitization [13]. To investigate relationships between fungal colonization in the house and sensitization to fungi, we measured levels of fungi in the houses of allergic children and serum IgE levels specific for fungi. We also conducted a questionnaire-based survey of daily life-styles to clarify the effects of house care in relation to fungi.

## Methods

This study was approved by the ethical committee of Tenri Hospital (Nara Prefecture, Japan). Subjects comprised 52 allergic children receiving medical follow-up at Tenri Hospital. Samples for the detection of fungi were obtained using 10 × 12-cm pieces of Tegaderm sticky tape (3M, St. Paul, MN) from 3 sites: bedding surface, house floor surface, and top of the television. Regarding house floors, 40 samples were collected from carpets, 8 from tatami mats, and the remainder from sofas (3 samples) and curtains (1 sample); because these 4 houses only had wood floorings that were unsuitable to collect samples using Tegaderm. We also collected fungi falling on the top of the television using Tegaderm placed on the television for 1 hour with the sticky side up. One child had no television, so a personal computer was used in place of a television. The procedure used to detect fungi was the same as described previously [14]. Briefly, Tegaderm samples were directly attached and cultured on potato-dextrose agar (PDA) and malt yeast 40% sucrose agar (M40YA) media. Samples from the bedding surface and floor surface were cultured on both PDA and M40YA media, and the result "detected" was given if a colony was found in either medium. Samples falling onto the television were cultured on PDA only.

Serum IgE levels to a combination of fungi (*Aspergillus, Alternaria, Cladosporium, Penicillium, Helminthosporium*, and *Candida*) were measured using UniCAP (Pharmacia Diagnostics AB, Uppsala, Sweden; renamed Phadia). Detectable levels of IgE (≥ 0.35 U_A_/ml) to a combination of fungi were seen in 32 children (positive group). The remaining 20 children were not sensitized to fungi (negative group). Median ages of the positive and negative groups were 8 years (range, 1-18 years) and 6 years (range, 3-18 years), respectively. Each IgE level specific for *Aspergillus, Alternaria, Cladosporium, Penicillium, Helminthosporium, Candida*, and *Mucor *was also measured using UniCAP in sera of the positive group.

We also conducted a questionnaire-based survey of daily lifestyles such as cleaning of the house, use of air purifiers, ventilation fans and heaters, placement of plants inside the house, and frequency of opening windows. This survey was conducted in the winter season between November and March. The guardians, mostly mothers, completed the questions.

### Statistical Analyses

A computer program, Statcel (OMS Publishing Inc., Saitama Prefecture, Japan), was used for statistical analyses. A χ^2 ^test was used for comparisons of fungus detection between groups. Spearman's correlation coefficient by rank test was used to identify correlations for IgE levels to each fungus. Mann-Whitney *U *test was used for comparisons of IgE levels to each fungus between groups. Mann-Whitney *U *test was also used for comparisons of daily lifestyles between positive and negative groups.

## Results

### Detection of Fungi in Houses

**Figure 1:**
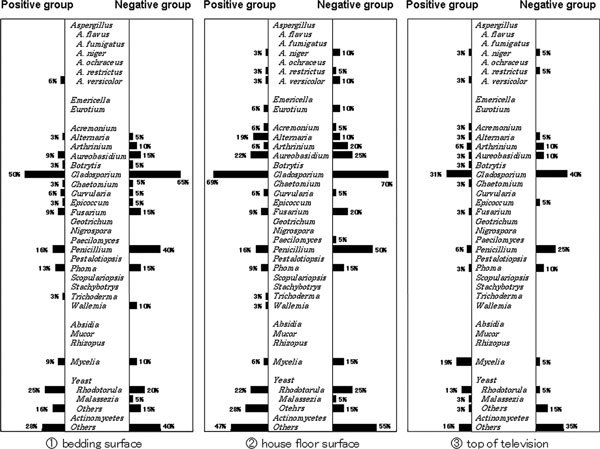
**Detection of fungi in houses**. Percentages of each fungus detected at the 3 sites are shown separately in the houses of positive and negative groups. *Penicillium *was more frequently detected on the floor surface in negative-group houses than in positive-group houses (χ^2 ^test; *P = *0.019), but in detection of the other fungi, no significant differences were seen between houses of positive and negative groups.

Percentages of each fungus detected at the 3 sites are shown separately in houses of the positive (32 houses) and negative groups (20 houses) (Figure 1). The pattern of the fungi detection was not markedly different at the 3 sites. *Cladosporium *was the most prevalent at all 3 sites. *Penicillium *was more frequently detected on the floor surface in negative-group houses than in positive-group houses (χ^2 ^test; *P *= 0.019), but in detection of the other fungi, no significant differences were seen between houses of positive and negative groups. In the 32 houses of the positive group, *Aspergillus *was found from 7 houses at any site, *Alternaria *from 8, *Cladosporium *from 27, *Penicillium *from 9, and *Mucor *from 0. The identification of *Helminthosporium *and *Candida *were not planned in this study. As only a few colonies, if any, of each fungus were found except for *Cladosporium*, numbers of colonies of each fungus were not evaluated.

### Detection of Each Fungus-Specific IgE

**Figure 2:**
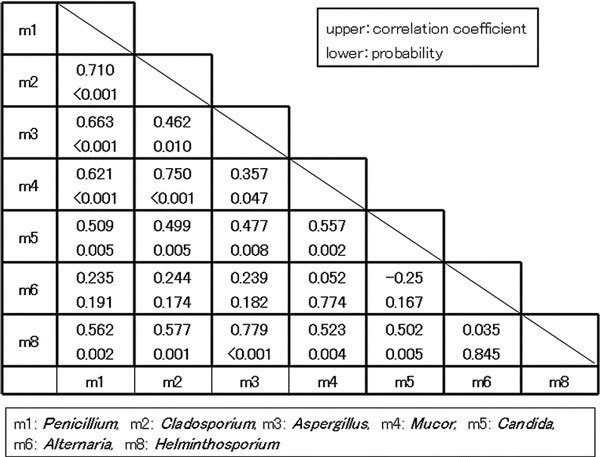
**Correlation between IgE levels for each fungus**. Correlations between IgE levels for each fungus were seen, except for *Alternaria*.

Numbers of serum samples with specific IgE level ≥ 0.70 U_A_/ml in the positive group were 10 for *Aspergillus*, 21 for *Alternaria*, 7 for *Cladosporium*, 12 for *Penicillium*, 5 for *Mucor*, 17 for *Helminthosporium*, and 14 for *Candida*. Correlations between IgE levels for each fungus were seen, except for *Alternaria *(Figure 2).

### Detection of Fungi in Houses and Fungus-Specific IgE Levels

**Figure 3:**
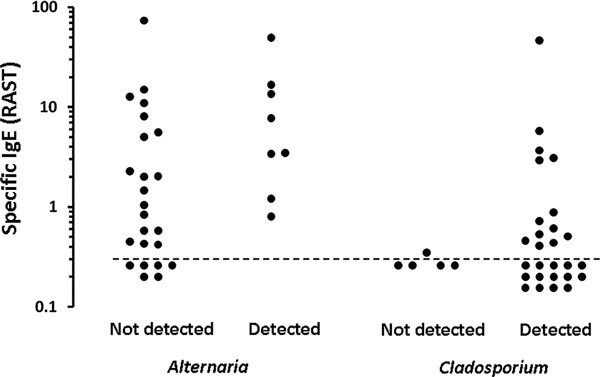
**Detection of *Alternaria *and *Cladosporium *in houses and specific IgE levels for each fungus**. Mann-Whitney *U *test was used for statistical evaluation (*P *= 0.033 for *Alternaria*; *P *= 0.145 for *Cladosporium*). The dashed line shows the limitation of IgE measurement (0.34 U_A_/ml).

Among the positive group, children in whose houses *Alternaria *was found showed higher levels of *Alternaria*-IgE than those in whose houses *Alternaria *was not found (Mann-Whitney *U *test; *P *0.033) (Figure 3). No such relationship was seen for any other fungi, including *Cladosporium *(Mann-Whitney *U *test; *P *= 0.145) (Figure 3).

### Questionnaires for Daily Lifestyles

**Figure 4:**
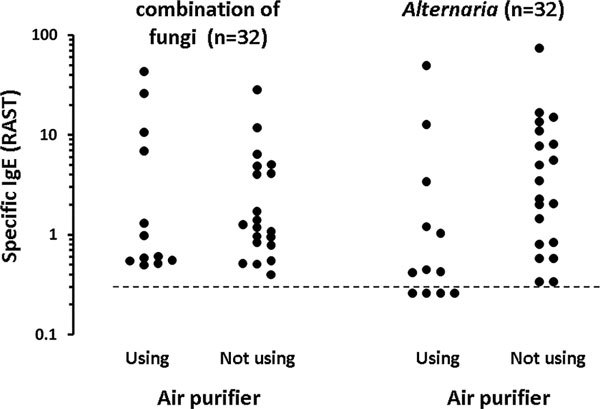
**Relationship between use of an air purifier and fungus-specific IgE levels**. Mann-Whitney *U *test was used for statistical evaluation (*P *= 0.786 for a combination of fungi; *P *= 0.039 for *Alternaria*). The dashed line shows the limitation of IgE measurement (0.34 U_A_/ml).

**Figure 5:**
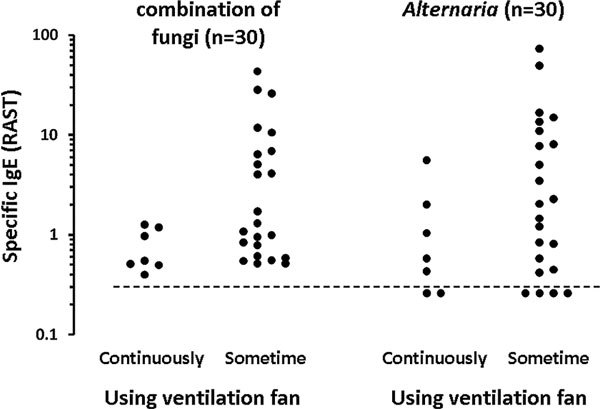
**Relationship between use of a ventilation fan and fungus-specific IgE levels**. Mann-Whitney *U *test was used for statistical evaluation (*P *= 0.022 for a combination of fungi; *P *= 0.140 for *Alternaria*). The dashed line shows the limitation of IgE measurement (0.34 U_A_/ml).

Among the positive group, *Alternaria*-IgE level was lower in children who were using an air purifier in the house than in children who were not using one (Mann-Whitney *U *test; *P *= 0.039) (Figure 4). No such an effect of an air purifier was seen for the other fungi-specific IgE or IgE to a combination of fungi (Mann-Whitney *U *test; *P *= 0.786). Among the positive group, IgE level specific for a combination of fungi was lower in children who were continuously using a ventilation fan than in children who were sometimes using one (Mann-Whitney *U *test; *P *= 0.022) (Figure 5). Such an effect of a ventilation fan was not seen in specific IgE levels for each fungus, including *Alternaria *(Mann-Whitney *U *test; *P = *0.140). Windows were less frequently opened for ventilation in houses of the positive group than in houses of the negative group (Mann-Whitney *U *test; *P *= 0.028) (Table 1). Conversely, no significant relationships were found between daily lifestyles and detection of fungi in houses.

**Table 1:** Frequency of Window Opening

Frequency of Window Opening	Negative Group	Positive Group	Total
Almost everyday	16	17	33
3-4 times/week	3	5	8
Once/week	1	6	7
Rarely	0	4	4
Total	20	32	52

Number of cases.

## Discussion

Both indoor and outdoor environments are important for allergic children. However, in this study, outdoor air was not sampled to determine levels of fungi, because outdoor levels were thought to be easily affected by weather, including wind and rain, and because outside was too vast to find sampling points. Fungi growth is also different in each area inside houses because of various conditions, including humidity and temperature, and therefore, samples to measure fungi were collected inside the houses at 3 sites (bedding, floor, and television). We selected these 3 sites because bedding is very close to children while they sleep for several hours a day; floor is also important where children stay for many hours in playing and walking; on the television, floating fungi which children inhale are dropping. As a result, only a few colonies, if any, of each fungus were found except for *Cladosporium*. Collecting samples from only 3 sites with small tapes were not enough for quantitative analyses.

The pattern of the fungi detection was not much different at the 3 sites. *Cladosporium *was the most prevalent fungus in the houses, as reported previously [15]. Houses of the positive and negative groups showed no marked differences in detection of fungi other than *Penicillium*. Samples were taken only once. Although seasonal changes in fungus detection in the indoor environment are reportedly small,[15] periodic measurement of fungi on multiple occasions in wider areas would have been preferable.

*Alternaria*-IgE was found most prevalently in the positive group. Many reports have described the relevance of *Alternaria *to allergic diseases [1-6]. Cross-reactivity is reportedly important [13] and in this study correlations of specific IgE levels between *Penicillium, Cladosporium, Aspergillus, Mucor, Candida*, and *Helminthosporium *were seen, but not between *Alternaria *and these fungi. Among the positive group, children in whose houses *Alternaria *was found showed higher levels of *Alternaria *IgE. The presence of *Alternaria *might strongly induce *Alternaria *IgE-sensitization, but why *Alternaria *IgE sensitization is unique in relation to other fungi remains unclear.

Use of an air purifier and continuous use of a ventilation fan seem to protect children against IgE sensitization to some fungi. In addition, frequent opening of windows for ventilation was seen in houses of children without sensitization to fungi. Opening windows may allow outdoor fungi to invade houses, but the ventilation may suppress fungi growth by drying indoor spaces when outdoor humidity is low. Because no significant relationships were noted in this study between daily lifestyles and detection of fungi in houses, more elaborate prospective study will be necessary to clarify the influence of daily home cleaning and maintenance practices on indoor fungal contamination and fungal IgE sensitization.

In our previous double-blind, controlled study, fungi were detected inside 1 of 10 HDM-free pillows and 6 of 10 common pillows after 1 year of use [12]. Even HDM-free pillows may be contaminated inside with fungi, as some spores are smaller than the mesh gap used in HDM-impermeable fabrics of HDM-free pillows. Care should be required regarding the presence of fungi in the indoor environment of allergic children.

## End Note

Sources of support: None.

Presented at the 43rd Annual Meeting of Japanese Society of Pediatric Allergy and Clinical Immunology, November 25, 2006, Chiba, Japan.
